# Case report: Ultrasonographic and computed tomographic imaging features of hemochromatosis in a dog

**DOI:** 10.3389/fvets.2023.1331392

**Published:** 2024-01-15

**Authors:** Mihyun Choi, Namsoon Lee

**Affiliations:** ^1^BON Animal Medical Center, Suwon, Republic of Korea; ^2^Section of Medical Imaging, Veterinary Medical Center, Chungbuk National University, Cheongju, Republic of Korea

**Keywords:** canine, iron-overload disorder, hemochromatosis, portal hypertension, pre-contrast CT

## Abstract

A castrated male mixed-breed dog weighing 7 kg presented with elevated liver enzymes and anorexia. Abdominal radiography revealed hepatomegaly with heterogeneous hepatic opacification, and abdominal ultrasonography showed a fine echotexture and heterogeneous parenchyma concurrent with a suspected acquired portosystemic shunt. Pre-contrast computed tomography (CT) showed marked hepatomegaly with homogeneous increased liver density and multiple enlarged abdominal lymph nodes with markedly increased parenchymal density. Histopathology of the hepatic and lymph node biopsy revealed accumulated abundant hemosiderin, and the Prussian Blue stain confirmed marked iron accumulation within the hepatocytes. Based on our review of the literature, this is the first case report describing the imaging diagnosis of hemochromatosis in a dog.

## Introduction

Iron is an essential micronutrient for hemoglobin synthesis, oxidation–reduction reactions, and cellular proliferation. The liver is the primary organ for iron storage and has the largest capacity to store excess iron (1). Several physiological mechanisms have developed to maintain iron homeostasis because too much iron can cause reactive oxygen species, potentially causing organ dysfunction. This condition is referred to as hemochromatosis (2). Iron overload diseases are classified as primary (genetic) or secondary (3, 4). Hereditary hemochromatosis is a recessive autosomal genetic disorder and presents the most common primary cause of iron overload disorders in humans (3); however, cases of hereditary hemochromatosis have not been reported in veterinary medicine. Secondary hemochromatosis includes increased iron absorption, anemia related to ineffective erythropoiesis, exogenous increase by ingestion, parenteral infusion, or multiple transfusions (4), and it is rarely reported in dogs (5–8). Common clinical signs of iron overload in humans include skin pigmentation, liver dysfunction, diabetes, and cardiomyopathy (3, 4). In veterinary medicine, presenting symptoms include gastrointestinal irritation, peripheral vascular collapse, pulmonary edema, and hepatic failure, as well as coma and death (1). The general methods for diagnosing hepatic iron overload in human medicine include computed tomography (CT), magnetic resonance imaging (MRI), or hepatic biopsies (9). However, to the best of our knowledge, there is no previous report of diagnostic imaging characteristics of hemochromatosis in dogs.

In our study, our patient presented with hepatic failure and was diagnosed using imaging modalities and a hepatic biopsy. This report aims to describe in detail the imaging features of hemochromatosis in a dog.

## Case description

A castrated male mixed-breed dog, weighing 7 kg, presented with a 2-week history of anemia, anorexia, soft stools, and elevated liver enzyme. Hepatic supportive care was administered at the referring veterinary hospital. The age of the dog was unclear, as the patient had been rescued 2 years earlier and stayed at an animal shelter. On physical examination, the dog showed cachexia (body condition score [BCS] 2/9). The body temperature, blood pressure, respiration rate, capillary refill time, and pulse were unremarkable. The complete blood count showed microcytic and normochromic anemia. Serum biochemistry revealed severely elevated liver enzymes (Table 1). Through the commercial assay, the patient was confirmed to have a Giardia infection, while other kits yielded negative results (SNAP 4Dx, IDEXX Laboratories Inc., Westbrook, ME; Rapid CDV, CIV, CPV, CCV, and Giardia Ag, Bionote, Hwaseong-si, South Korea for detecting antigen of canine distemper, influenza, parvovirus, coronavirus, and Giardia).

**Table 1:** Hematological and biochemical analyses in a dog with hemochromatosis.

	Day 0	Day 30	Unit	Reference range
WBC	30.30	40.62	K/ul	5.05–16.76
RBC	5.47	5.73	M/ul	5.65–8.87
HGB	11.7	11.8	g/dl	13.1–20.5
HCT	30.9	31.2	%	37.3–61.7
MCV	56.5	54.5	fL	61.6–73.5
MCHC	37.9	37.8	g/dL	32.0–37.9
PLT	286	427	K/ul	148–484
PT		16.5	sec	14–19
APTT		68.9	sec	75–105
TP	6.9	6.9	g/dl	5–7.2
ALB	2.7	3	g/dl	2.6–4.0
ALP	>3,500	>3,500	U/L	47–254
ALT	257	377	U/L	17–78
AST	137	271	U/L	17–44
GGT	102	82	U/L	5–14
T-CHOL		171	mg/dl	111–312
TBIL	0.6	0.6	mg/dl	0.1–0.5
BUN	30.3	29.6	mg/dl	9.2–29.2
CREA	0.68	0.42	mg/dl	0.4–1.4
NH3		247	Umol/L	16–75
GLU	127	147	mg/dl	60–115
CRP	16	9	mg/l	1–9

WBC, white blood cell; RBC, red blood cell; HGB, hemoglobin; HCT, hematocrit; MCV, mean corpuscular volume; MCHC, mean corpuscular hemoglobin concentration; PLT, platelet; PT, prothrombin time; APTT, activated prothrombin time; TP, total protein; ALB, albumin; ALP, alkaline phosphatase; ALT, alanine aminotransferase; AST, aspartate aminotransferase; GGT, gamma-glutamyl transferase; T-CHOL, total cholesterol; TBIL, total bilirubin; BUN, blood urea nitrogen; CREA, creatinine; NH3, ammonia; GLU, glucose; CRP, C-reactive protein.

**Figure 1:**
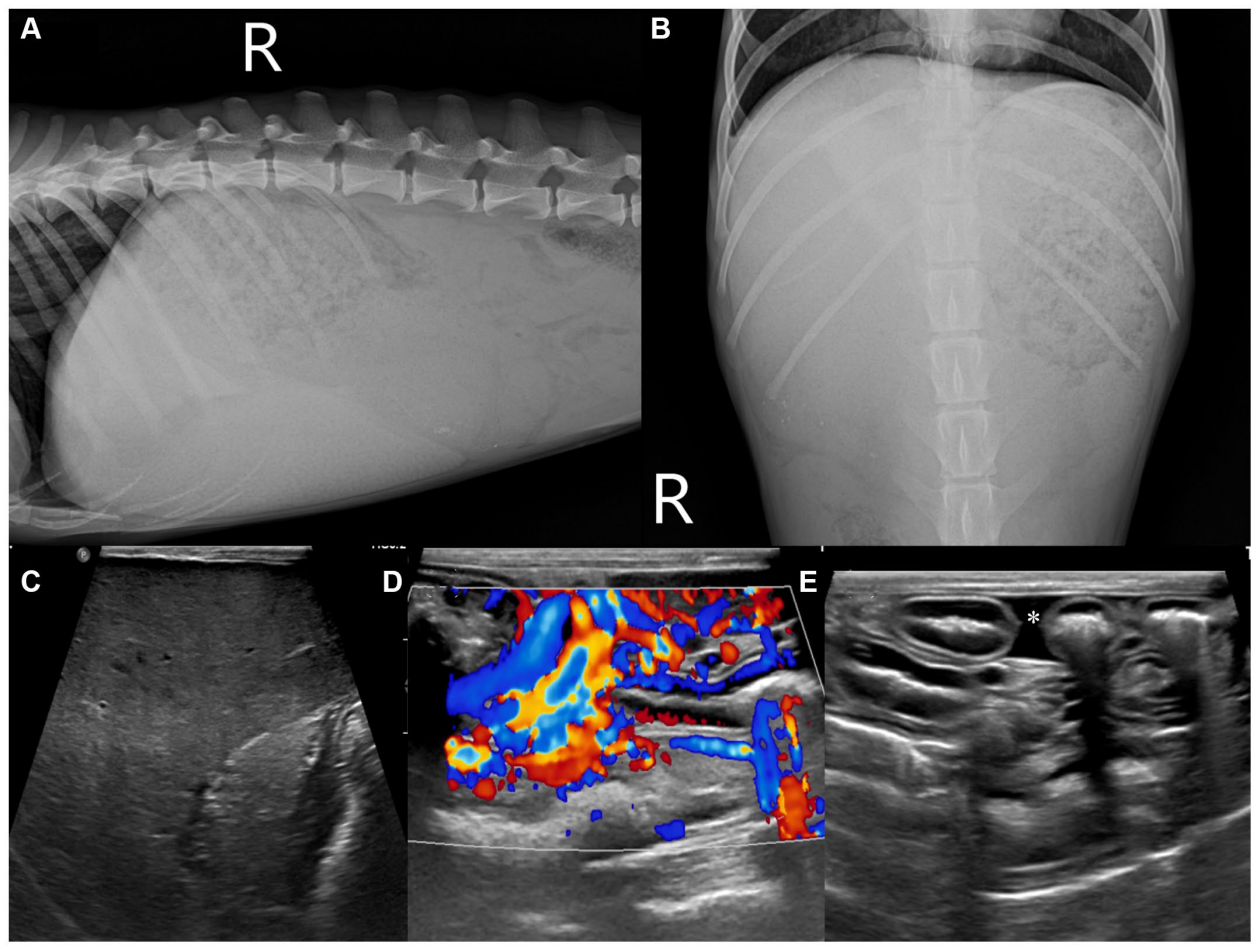
Abdominal radiographic and ultrasonographic examination **(A–E)**. Right lateral **(A)** and ventrodorsal **(B)** radiographs of the abdomen. The caudoventral liver margins are rounded, and the gastric axis is deviated caudally. The liver parenchyma shows an irregular net-like, heterogeneously increased opacity. B-mode and color Doppler ultrasonographic images of the liver **(C)**, near the left kidney **(D)**, and peritoneal cavity **(E)**. The hepatic parenchyma illustrates a uniformly increased echogenicity with reduced visualization of the portal structures **(C)**. Numerous small tortuous vessels were observed near the left kidney **(D)** and accumulated a small amount of peritoneal fluid **(E)**, asterisk.

Abdominal radiography (1417WGC, Rayence Co., Ltd., Hwaseong-si, South Korea) was performed. Severe hepatomegaly with serosal detail loss was observed on the lateral view. The hepatic opacity was heterogeneously increased net-like (Figures 1A,B). The thoracic radiographs were unremarkable. Abdominal ultrasound was performed using 4–18 MHz linear transducers (EPIQ 7 ELITE, Philips Healthcare, Amsterdam, The Netherlands). The result showed an enlarged liver with a uniformly increased echogenicity, fine echotexture, heterogeneous parenchyma (Figure 1C), increased echogenicity of the gall bladder wall without acoustic shadowing, multiple small tortuous vessels near the left renal and splenic regions with a small amount of peritoneal fluid (Figures 1D,E), and multiple diffuse hypoechoic nodular lesions in the spleen. No other remarkable findings in the peritoneal cavity were observed on ultrasound. The differential diagnoses for the hepatic lesions included cholangiohepatitis or, less likely, a malignant tumor, leading to an acquired portosystemic shunt. The splenic lesion may have presented secondary to the anemia due to extramedullary hematopoiesis. The tentative diagnosis was cholangiohepatitis concurrent with mild secondary chronic anemia due to intestinal hemorrhage caused by Giardia infection. The patient received hepatic support (including ursodeoxycholic acid (UDCA), 10 mg/kg, twice a day; silymarin, 10 mg/kg, twice a day) and an antiprotozoal agent (metronidazole, 10 mg/kg, twice a day). However, the dog showed no signs of improvement (Table 1), and repeat abdominal ultrasonography showed no changes at 1-month follow-up. Multiphase helical CT was then performed after 1 month of treatment for further investigation.

The CT was performed using a 64-slice scanner (Aquillion64™, Toshiba Medical Systems, Tochigi, Japan). Anesthesia was induced with propofol (6 mg/kg, IV) and maintained with isoflurane. The CT protocol was as follows: 0.5 mm slice thickness, 120 kVp, 150 mAs, 512 × 512 matrix, and 0.75 s/rotation. Iohexol (Omnipaque™ 300, GE Healthcare, Chicago, IL, USA) was used for intravenous contrast CT studies at a dose of 700 mgI/kg, administrated by a power injector (Dual Shot GX-7, Nemoto Kyorindo co., Ltd., Tokyo, Japan) with an injection rate of 2.5 m/s. A bolus-tracking protocol was used during the arterial acquisition in a cranial to caudal direction and triggered when the aorta that was just caudal to the porta hepatis was reached at 250 HU, demonstrating the arterial phase. According to the standard procedure, the portal phase was acquired in a cranial to caudal direction 40 s after contrast administration. The delayed phase was acquired after 120 s, and the images were reconstructed in 2.0 mm transverse sequences, with sagittal, dorsal, and oblique reformatted images using soft tissue and lung algorithms.

**Figure 2:**
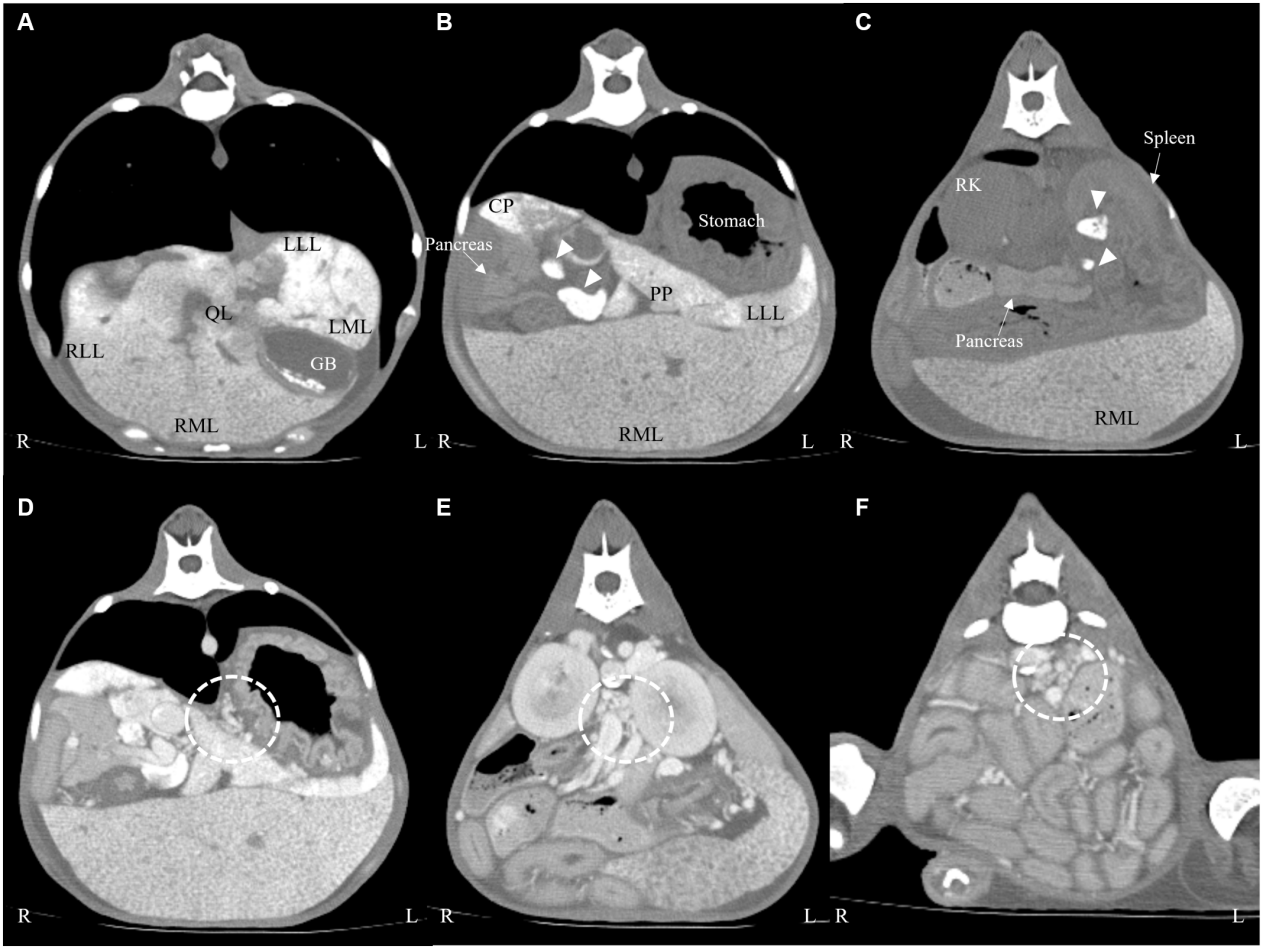
Transverse computed tomography images: pre-contrast **(A–C)** and post-contrast **(D–F)** images on a soft tissue algorithm. Generalized liver **(A–C)** and lymph node (**(B,C)**, arrow heads) enlargement with increased attenuation compared with the splenic parenchyma. There were numerous varices in the left gastric vein **(D)**, left renal **(E)**, and colic region **(F)** (dotted line). LLL, left lateral lobe; LML, left medial lobe; RLL, right lateral lobe; RML, right medial lobe; QL, quadrate lobe; CP, caudate process of caudate lobe; PP, papillary process of caudate lobe; GB, gall bladder; RK, right kidney.

On pre-contrast imaging, marked hepatomegaly was observed, except for the left hepatic lobe with the gall bladder abnormally deviated to the left. The hepatic parenchyma had a mottled-like appearance. The homogeneous increased parenchymal density was observed on all the liver lobes (Figures 2A–C). The left hepatic lobe was small, with a more prominent homogeneous increased parenchymal density (Figure 2A). The hepatic, splenic, and gastric lymph nodes were enlarged with marked increased parenchymal density, as observed in the pancreatic parenchyma (Figures 2B,C). For quantitative evaluation, three different circular regions of interest were drawn over the left and right liver parenchyma, lymph nodes, and pancreatic parenchyma, avoiding major vessels. For each region of interest, the mean (standard deviation, [SD]) attenuation values (HU, Hounsfield Units) were recorded, and an average of the three measurements was calculated. Attenuation values of the splenic parenchyma were also recorded to compare them with the hepatic, lymph nodal, and pancreatic values. The mean (SD) left and right hepatic, lymph nodal, pancreatic parenchymal, and splenic attenuation values were 254.26 HU (17.28), 129.21 HU (14.96), 264.75 HU (7.48), 71.36 HU (3.67), and 52.95 HU (3.18), respectively. On post-contrast images, there were numerous varices in the esophagus, left gastric vein, pancreaticoduodenal vein, left renal to splenic veins, and the mesenteric and colic regions (Figures 2D–F). There was a small amount of accumulated peritoneal fluid. No abnormal lesions were detected in the thorax.

Based on the clinical and imaging findings, the differential diagnosis was atypical infiltration (e.g., heavy metal accumulation) of the hepatic parenchyma and lymph nodes and, less probably, neoplasia with induced portal hypertension and a consequential acquired portosystemic shunt.

A laparoscopic hepatic parenchymal and lymph node biopsy was performed (Stryker 1,288 HD, Stryker, Chicago, IL, USA). A coagulation panel showed a mildly delayed partial prothrombin time (124.5 s; reference range, 75–105 s). Anesthesia was induced with propofol (6 mg/kg, IV) and maintained using isoflurane with an oxygen supply, and the dog was appropriately monitored. A two-port technique was used. The patient was placed in dorsal recumbency, and a 6-mm subumbilical camera port was positioned on the ventral midline just caudal to the umbilicus using the modified Hasson technique. A second 6-mm instrument port was placed midway between the camera port and the xiphoid process. A 5-mm, 30-degree laparoscope was inserted, and before obtaining any biopsy sample, the diaphragmatic and visceral surfaces of each liver lobe were evaluated for macroscopic lesions. A 5-mm cup biopsy instrument was introduced, and six samples were obtained (including 2 right middle, 1 right lateral, 1 left medial, 1 quadrate liver lobes, and gastric lymph node). The tissues from the hepatic parenchyma and lymph node were fixed in 10% neutral-buffered formalin and sent for histological evaluation at a laboratory (IDEXX Laboratories Inc., Westbrook, ME, USA).

**Figure 3:**
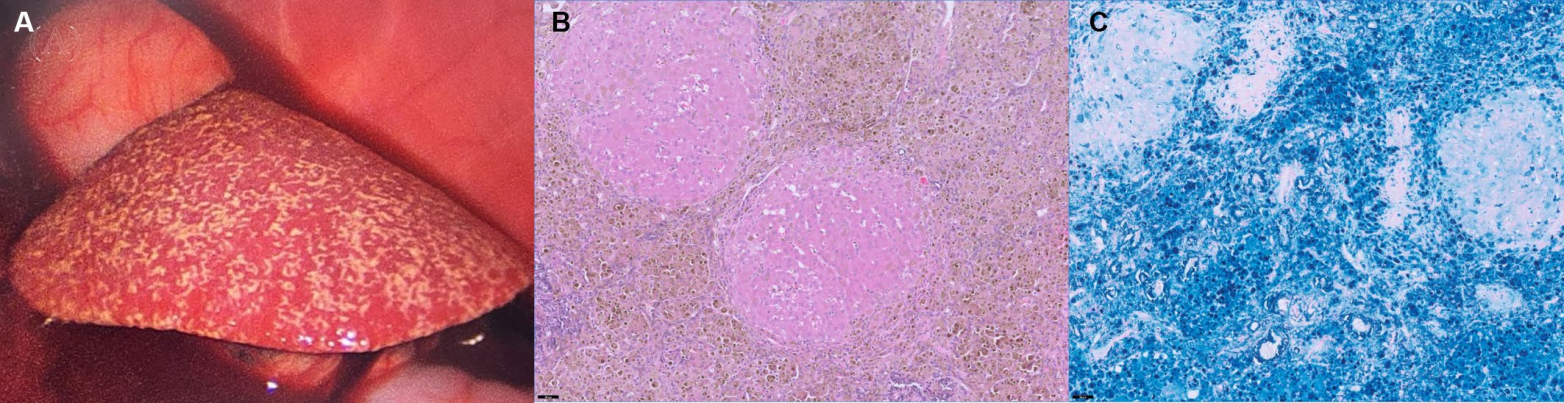
Laparoscopic photography **(A)** of the liver and histopathological findings after HE staining **(B)** and Prussian Blue staining **(C)** of the liver. The liver was diffusely yellow to tan and showed multifocal micro-nodulation macroscopically **(A)**. There were marked abundant Kupffer cell clusters containing intracytoplasmic hemosiderin (**B**, ×20), and Prussian Blue stain (**C**, ×20) confirmed marked iron accumulation within the hepatocytes.

On macroscopic evaluation, the liver was diffusely yellow to tan and enlarged with multifocal to coalescing micro-nodulations (Figure 3A). After the biopsy, the patient was closely monitored for hemorrhage. Although an increased amount of peritoneal fluid was confirmed 1 day after the biopsy procedure, there were no signs of hemodynamic instability or relevant decrease in hematocrit (pre-biopsy hematocrit; 27.7%, hematocrit 1 day after biopsy; 23.6%; and reference range, 37.3–61.7%). Fluid analysis confirmed that the ascites cause by ‘no’ bleeding (hematocrit, 0.8%). A presumptive diagnosis of chronic hepatopathy was made on the macroscopic findings, and thus, the patient received hepatic supportive care (UDCA, 10 mg/kg, bid; silymarin, 10 mg/kg, bid; SAMe, 10 mg/kg, bid; doxycycline 5 mg/kg, bid and spironolactone, and 1 mg/kg, bid) and albumin transfusion. However, the symptoms progressed to more severe anemia and hypoalbuminemia, and the patient eventually died 7 days after the laparoscopic biopsy.

Histological examination of the liver biopsy samples confirmed marked vascular mural mineralization or calcification with portal vascular reduplication (Figure 3B). Throughout the affected portal interstitium, there were marked abundant Kupffer cell clusters containing intracytoplasmic hemosiderin. Prussian Blue staining showed marked iron accumulation within the hepatocytes in the regenerative nodules, interstitial septa, and groups of macrophages (Figure 3C). Rhodanine stain showed no pathologic copper accumulation. Gastric lymph nodes also showed very similar changes to those observed in the liver. A definitive diagnosis of hemochromatosis-induced hepatic dysfunction, and subsequent portal hypertension was made based on the CT and histopathological examination.

## Discussion

Hemochromatosis is defined as a disorder characterized by excessive iron deposition, leading to multiple organ dysfunction, with a particular emphasis on liver toxicity. Serum ferritin concentrations, which are associated with tissue iron storage and provide a useful measure of total body iron, are used to diagnose iron overload conditions (9). However, because ferritin is an acute-phase protein, animals with coexisting inflammation, as well as those with other illnesses such as hemolytic and hepatic diseases and some neoplastic disorders, may have higher quantities of ferritin (1).

In human medicine, hepatic biopsy and imaging study (CT or MRI) are generally the diagnosing methods. Ultrasound is not a suitable modality for evaluating hepatic iron overload, as it cannot detect iron deposition (10). Unenhanced CT shows a homogeneous increase in hepatic parenchymal attenuation. In humans, the unenhanced CT attenuation values of the normal liver vary from 50 to 65 HU (vs. a range of 60–70 HU for dogs) (11). In the cases of human hepatic iron overload, unenhanced CT reveals increased hepatic attenuation (above 70 HU) (12) because of the greater electron density associated with iron atoms compared with normal liver tissue. Increased hepatic attenuation may be observed in storage disorders, sarcoidosis, copper hepatopathy, and drug-induced (amiodarone, methotrexate, and gold) hepatotoxicity, thus caution is required during interpretation (13–15). When it comes to diagnosing iron overload, CT has a low sensitivity of 63% and a high specificity of 96% (13). In our case, abdominal ultrasound did not provide supportive evidence for diagnosing hemochromatosis, but it did reveal distinctions from general hepatic disease. The liver showed a prominent enlarged and hyperechoic parenchyma with multiple acquired portosystemic shunts. We suspected hepatic causes of portal hypertension but the initial diagnosis was puzzling. In addition, despite the absence of a report related to iron overload on CT examination in veterinary medicine, our case showed results similar to those in human medicine.

Magnetic resonance imaging (MRI) is the most common non-invasive tool in human medicine for diagnosing iron overload in the liver, determining the severity, and monitoring with high sensitivity, specificity, and positive and negative predictive values (16). The iron ions have superparamagnetic properties, which cause shortening of T1, T2, and T2* relaxation times and result in a hypointense signal in the affected organs (16). Gradient echoes are more sensitive to susceptibility effects than spin echoes, and thus, mild cases of iron overload may be apparent only on gradient echo imaging (17). If iron overload is severe, the degree of signal loss may be marked on all images (16). We were unable to conduct an MRI examination on our patient, highlighting the need for further studies in veterinary medicine regarding diagnosing and monitoring hepatic iron overload disease.

Generally, iron is first delivered to the reticuloendothelial system (RES), and when the RES storage capacity is saturated, iron accumulates in hepatocytes and parenchymal cells of other organs (18). Therefore, in human medicine, iron overload can be categorized as reticuloendothelial, parenchymal, or renal deposition patterns, which can help distinguish the potential causes of hemochromatosis. First, the reticuloendothelial deposition pattern, which happens secondary to repeated transfusions, generally occurs in the reticuloendothelial system (e.g., the liver, spleen, and bone marrow) and is not associated with tissue damage (19, 20). Second, the parenchymal deposition pattern develops in cases of increased iron absorption, such as primary hemochromatosis or chronic anemia with inefficient erythropoiesis. The excess iron accumulates, leading to tissue damage initially in the periportal hepatocytes and spreading to the remaining portion of the liver, pancreas, and thyroid gland (20, 21). Third, a renal deposition pattern is only observed in the instance of intravascular hemolysis in the renal cortex (22). Our patient showed a parenchymal deposition pattern (e.g., the liver, lymph node, and pancreas), and the cause of the hemochromatosis was thus suspected to be primary or chronic anemia. Although the patient had mild microcytic, normochromic anemia, it was deemed a consequence of the disease (chronic hepatic disease) rather than the underlying cause such as iron deficiency state. In addition, conditions such as pyruvate kinase deficiency can cause severe chronic or intermittent hemolytic anemia. However, our patient did not show hemolytic anemia at presentation and had no anemic status when it was rescued. Therefore, the cause of the hemochromatosis remains unclear.

In humans, hepatomegaly is observed frequently in the early stages, but cirrhosis occurs when the hemochromatosis is progressive (9). Histologically, as iron accumulates, fibrosis slowly develops in a periportal distribution. Eventually, portal–portal bridging fibrosis and cirrhosis occur, resulting in a diffuse micronodular pattern (2), which induces increased portal vein blood flow resistance and leads to portal hypertension. In our patient, the liver size was uneven, with the left liver lobe notably smaller and the other lobes enlarged. The left liver lobe (254 HU) showed a higher HU value than the other liver lobes (129 HU). Initially, we suspected that the left liver had the most iron accumulation and cirrhotic change, but histopathologic features that were compatible with chronic active hepatitis were lacking. In human medicine, the correlation between CT attenuation and hepatic iron concentration is poor (13), which needs caution for interpretation. In addition, our case was considered to be hemochromatosis-inducing portal hypertension, irrespective of the stage. However, the cause of the variation in hepatic size remains unknown.

To prevent irreversible organ damage in patients with hemochromatosis, early diagnosis and prompt initiation of appropriate treatments are critical (9). Phlebotomy is the standard treatment in humans. However, in the cases of both iron overload and anemia, iron chelation therapy may also be considered (9). However, as hemochromatosis has rarely been reported in veterinary medicine, there is no experience with iron chelation in dogs. Additionally, since the patient had already progressed to portal hypertension, conservative therapy was selected but it eventually led to death.

Our study had some limitations. First, an MRI of the liver and serum ferritin level tests were not performed due to financial limitations since our patient was an animal shelter rescue. However, CT and histopathologic examination confirmed the diagnosis of hemochromatosis. Second, the cause of hemochromatosis is unclear. Although the imaging pattern suggested parenchymal iron deposition, a criterion adapted from human medicine, other causes need to be ruled out. Finally, patient history including factors such as repeated blood transfusions and excessive iron administration was unknown, and a necropsy was not performed.

In conclusion, hemochromatosis should be included in the clinical differential diagnosis lists of dogs with hepatic dysfunction concurrent with hepatomegaly and portal hypertension. Since abdominal ultrasound is not a suitable modality for evaluating hepatic iron overload, multimodal imaging examinations should be performed to investigate the condition. Due to a notably elevated HU value of the hepatic parenchyma on pre-contrast images, CT enabled both qualitative and quantitative diagnosis of diffuse iron overload status and was confirmed by histopathology for a definitive diagnosis.

## Data availability statement

The original contributions presented in the study are included in the article/supplementary material, further inquiries can be directed to the corresponding author.

## Ethics statement

Ethical approval was not required for the study involving animals in accordance with the local legislation and institutional requirements because Ethical approval was not required for the study because the case report is a description of a clinical case. Written informed consent could not obtained because the patient is rescue dog, however permitted diagnostic test from animal protection center in Suwon.

## Author contributions

MC: Conceptualization, Formal analysis, Writing – original draft. NL: Supervision, Writing – review & editing.
